# Regulation of Sulfate Uptake and Assimilation in Barley (*Hordeum vulgare*) as Affected by Rhizospheric and Atmospheric Sulfur Nutrition

**DOI:** 10.3390/plants9101283

**Published:** 2020-09-28

**Authors:** Ties Ausma, Luit J. De Kok

**Affiliations:** Laboratory of Plant Physiology, Groningen Institute for Evolutionary Life Sciences, University of Groningen, 9747 AG Groningen, The Netherlands; l.j.de.kok@rug.nl

**Keywords:** APS reductase, air pollution, HvST1, hydrogen sulfide, sulfate deficiency, sulfate transporter

## Abstract

To study the regulation of sulfate metabolism in barley (*Hordeum vulgare*), seedlings were exposed to atmospheric hydrogen sulfide (H_2_S) in the presence and absence of a sulfate supply. Sulfate deprivation reduced shoot and root biomass production by 60% and 70%, respectively, and it affected the plant’s mineral nutrient composition. It resulted in a 5.7- and 2.9-fold increased shoot and root molybdenum content, respectively, and a decreased content of several other mineral nutrients. Particularly, it decreased shoot and root total sulfur contents by 60% and 70%, respectively. These decreases could be ascribed to decreased sulfate contents. Sulfate deficiency was additionally characterized by significantly lowered cysteine, glutathione and soluble protein levels, enhanced dry matter, nitrate and free amino acid contents, an increased APS reductase (APR) activity and an increased expression and activity of the root sulfate uptake transporters. When sulfate-deprived barley was exposed to 0.6 µL L^−1^ atmospheric H_2_S, the decrease in biomass production and the development of other sulfur deficiency symptoms were alleviated. Clearly, barley could use H_2_S, absorbed by the foliage, as a sulfur source for growth. H_2_S fumigation of both sulfate-deprived and sulfate-sufficient plants downregulated APR activity as well as the expression and activity of the sulfate uptake transporters. Evidently, barley switched from rhizospheric sulfate to atmospheric H_2_S as sulfur source. Though this indicates that sulfate utilization in barley is controlled by signals originating in the shoot, the signal transduction pathway involved in the shoot-to-root regulation must be further elucidated.

## 1. Introduction

Barley (*Hordeum vulgare*) is cultivated globally for the production of alcoholic beverages and animal fodder [1,2]. Since the crop is frequently grown on light textured soils, it is prone to sulfur (S) deficiency [1]. S is an indispensable macronutrient for plants that is needed for the synthesis of proteins and other organic compounds [3]. An insufficient S supply may limit endosperm modification during malting by lowering the grain’s content of, e.g., *B*- and *D*-hordein proteins [1,2]. Moreover, it may lower kernel *S*-methylmethionine (SMM) and dimethylsulfoxide (DMSO) levels, which are the precursors for dimethylsulfide (DMS) synthesis [1]. DMS decisively affects the aroma, taste and flavor of beverages [1,2].

Plants generally acquire S as sulfate via the roots [3]. In shoot and root plastids, sulfate is activated to adenosine 5’-phosphosulfate, which is next reduced to sulfite by APS reductase (APR) [3]. Sulfite is subsequently reduced to sulfide, which is incorporated in cysteine by the cysteine synthase complex [3]. This incorporation couples S assimilation to nitrogen (N) assimilation [3]. From cysteine, numerous S-containing organic compounds can be synthesized [3].

The rate of sulfate metabolism into cysteine, which is tuned to the plant’s S demand for growth, is controlled by the activity of the root sulfate uptake transporters and APR [3]. Thus, sulfate deprivation generally enhances the expression of the root sulfate transporters, the root’s sulfate uptake capacity and the expression and activity of APR [3].

Atmospheric hydrogen sulfide (H_2_S) is potentially phytotoxic, though species differ considerably in their H_2_S susceptibility [4]. Whereas prolonged exposure to 0.03 μL L^−1^ H_2_S (a realistic level for industrially- and agriculturally polluted areas) inhibited the biomass production of sensitive dicot species, monocot species, including barley, are rather H_2_S insusceptible [4]. These species can tolerate up to 1.5 μL L^−1^ H_2_S, without negative effects on plant biomass production [4,5]. In monocots, the meristem is sheltered by leaves. Therefore, H_2_S can hardly penetrate the meristem, which may explain the H_2_S tolerance of these species [4,5].

Plants can use H_2_S, absorbed by the foliage, as a S source for growth [4,6,7]. Plants may even grow with atmospheric H_2_S as the sole S source (viz. when no sulfate is supplied to the root) [4,6]. H_2_S fumigation of sulfate-deprived plants may fully alleviate the development of S deficiency symptoms. In the many tested plant species, the rate of foliar H_2_S uptake followed Michaelis–Menten kinetics with respect to the atmospheric H_2_S level [8]. The kinetics are controlled by the rate of H_2_S incorporation into cysteine and subsequently other molecules [4,8]. Typically, shoot cysteine content and that of the tripeptide glutathione increase significantly upon H_2_S fumigation, indicating that absorbed H_2_S is metabolized with high affinity in these thiols [4].

The fumigation of plants with H_2_S is a powerful way to obtain insights into the regulation of sulfate uptake and assimilation [4]. Atmospheric H_2_S is directly incorporated in cysteine in shoots and, consequently, studying the impact of H_2_S fumigation on the sulfate transporters and APR, in the presence and absence of a sulfate supply, may reveal if these enzymes are regulated by signals originating in the shoot or root environment [4].

Thus, in the current research, the interaction between atmospheric H_2_S and rhizospheric sulfate nutrition was analyzed in barley. Plants were H_2_S fumigated in the presence and absence of a sulfate supply. The mineral nutrient composition, S and N metabolite content, APR activity, and the expression and activity of the root sulfate transporters were analyzed.

## 2. Results and Discussion

### 2.1. Impact of Sulfate Deprivation and H_2_S Fumigation on Biomass Production and Mineral Nutrient Content

**Figure 1:**
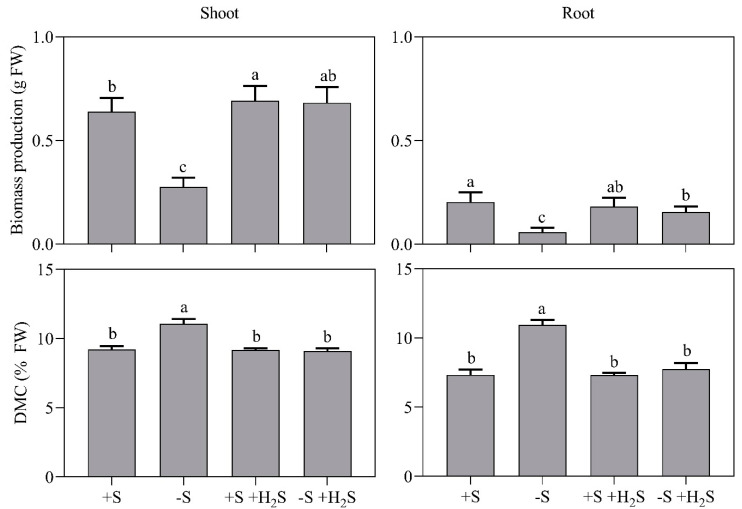
Biomass production and dry matter content (DMC) of barley shoots and roots in the presence and absence of a H_2_S and sulfate supply. Seven-day old seedlings were grown on a 50% Hoagland nutrient solution at 1 mM (+S) or 0 mM sulfate (−S) and exposed to 0 or 0.6 µL L^−1^ H_2_S for 7 days. The initial shoot and root weights were 0.16 ± 0.01 and 0.08 ± 0.01 g FW, respectively. Data on biomass production represent the average of 2 experiments with 12–13 measurements with 3-6 plants per measurement (±SD). Data on DMC represent the average of 3 measurements with 6 plants per measurement (±SD). Significant differences between treatments are indicated with different letters (*P* ≤ 0.05; two-way ANOVA; Tukey’s HSD test as a post-hoc test).

A 7-day sulfate deprivation reduced shoot and root biomass production by 60% and 70%, respectively (Figure 1). Since shoot and root biomass production were similarly reduced, sulfate deprivation hardly affected the shoot-to-root ratio. Shoot and root dry matter contents were enhanced by sulfate deprivation by 1.2-fold and 1.5-fold, respectively, which may be due to an increased level of non-structural carbohydrates (viz. sugars and starch; Figure 1) [3].

Exposure of sulfate-sufficient plants to 0.6 µL L^−1^ atmospheric H_2_S hardly affected biomass production and dry matter content (Figure 1). However, upon sulfate deprivation, H_2_S fumigation alleviated the establishment of S-deficiency signs. The biomass production and dry matter content of sulfate-deprived H_2_S-fumigated plants were comparable to those of sulfate-sufficient plants (Figure 1). Obviously, in accordance with previous observations [6], barley could use H_2_S, absorbed by the foliage, as a S source for the synthesis of organic compounds.

Sulfate deprivation resulted in a strong (60%) decrease in the shoot’s total S content, whereas the shoot’s content of most other mineral nutrients was hardly affected (Table 1). In the shoots of sulfate-deprived plants, N content was slightly decreased (by 10%), whereas P and Mo contents were increased by 1.3- and 5.7-fold, respectively. By contrast, sulfate deprivation significantly affected the root’s mineral nutrient composition (Table 1). It decreased S, P, K, Mn, Cu and Zn contents by 70%, 30%, 20%, 50%, 40% and 40%, respectively, and it increased Mo content by 2.9-fold.

H_2_S fumigation of sulfate-sufficient plants did, apart from a 1.6-fold increased shoot S level, not result in changes in the mineral nutrient composition (Table 1). H_2_S fumigation of sulfate-deprived plants alleviated the impacts of sulfate deprivation on shoot S, N and P contents and on shoot and root Mo contents (Table 1). By contrast, H_2_S fumigation hardly alleviated the other impacts of sulfate deprivation on the plant’s mineral nutrient composition (Table 1). Clearly, similar to observations in other plants [7,9,10], in barley there is no strict coupling between the metabolism of S and the majority of other mineral nutrients.

### 2.2. Impact of Sulfate Deprivation and H_2_S Fumigation on Sulfur and Nitrogen Metabolite Content

**Figure 2:**
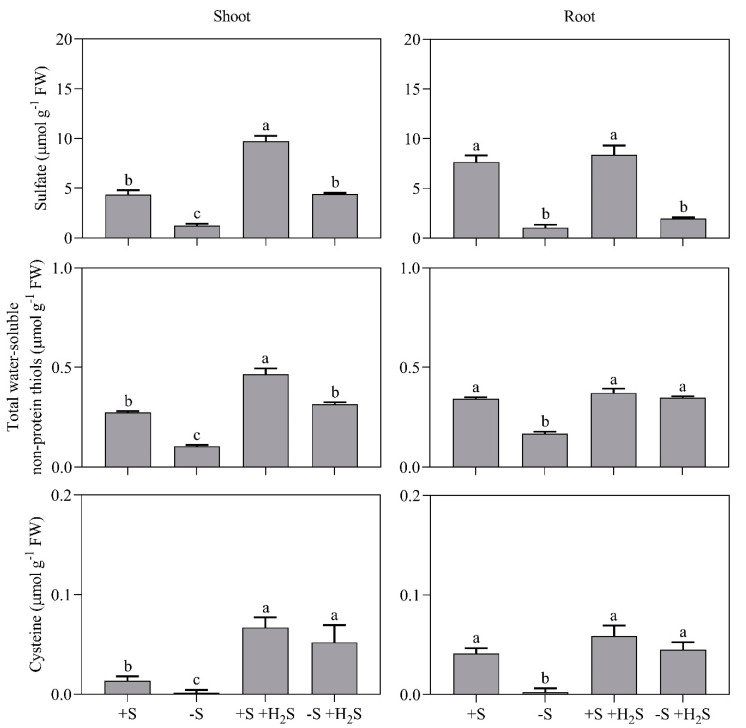
Sulfate, total water-soluble non-protein thiol and cysteine content of barley shoots and roots in the presence and absence of a H_2_S and sulfate supply. For experimental details, see the legend of Figure 1. Data represent the average of 3 measurements with 3 plants per measurement (±SD). Significant differences between treatments are indicated with different letters (*P* ≤ 0.05; two-way ANOVA; Tukey’s HSD test as a post-hoc test).

The observed changes in total S content upon sulfate deprivation and H_2_S fumigation could, at least partly, be attributed to changes in sulfate content. Sulfate deprivation resulted in a 70 and 90% decrease in the shoot and root sulfate content, respectively (Figure 2). H_2_S fumigation alleviated the decrease in shoot sulfate content (Figure 2). Moreover, H_2_S fumigation of sulfate-sufficient plants enhanced shoot sulfate levels by 2.2-fold (Figure 2).

The (total water-soluble non-protein) thiol pool represented a minor proportion of total S in the plant (approximately 3%; Figure 2). Sulfate deprivation decreased shoot and root thiol levels by 40% and 50%, respectively (Figure 2). These changes could mainly be attributed to changes in glutathione content, since cysteine accounted for only 5% and 10% of the thiol pool in sulfate-sufficient shoots and roots, respectively (Figure 2). Yet, sulfate deprivation decreased shoot and root cysteine levels by almost 100% (Figure 2). H_2_S fumigation alleviated the decreases in thiol levels and it even increased shoot cysteine levels by 3.9-fold (Figure 2). Moreover, H_2_S fumigation of sulfate-sufficient plants increased the total water-soluble non-protein thiol and cysteine level of the shoot by 1.7- and 5.0-fold, respectively (Figure 2). Evidently, H_2_S is with high affinity assimilated into cysteine and subsequently glutathione.

**Figure 3:**
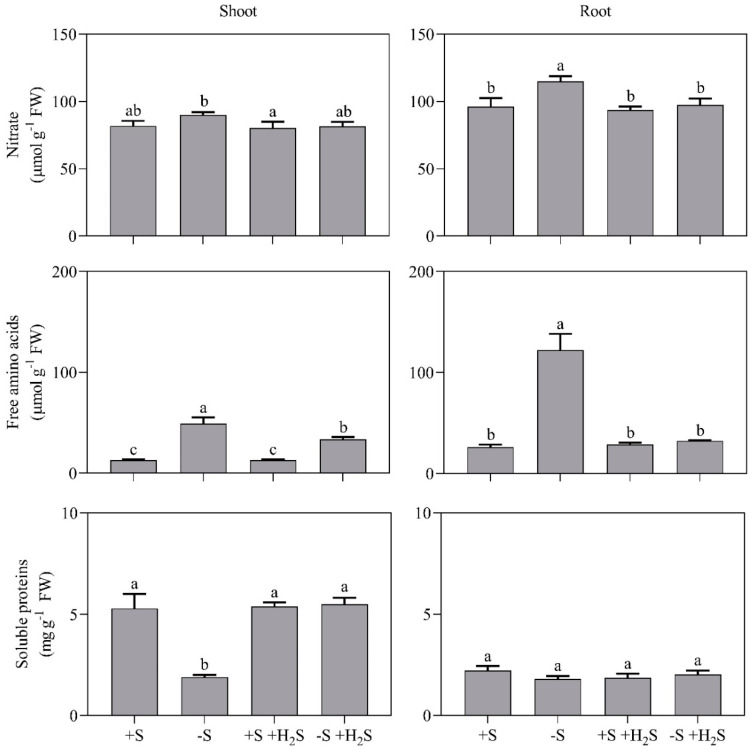
Nitrate, free amino acid and soluble protein content of barley shoots and roots in the presence and absence of a H_2_S and sulfate supply. For experimental details, see the legend of Figure 1. Data represent the average of 3 measurements with 3 plants per measurement (±SD). Significant differences between treatments are indicated with different letters (*P* ≤ 0.05; two-way ANOVA; Tukey’s HSD test as a post-hoc test).

Sulfate deprivation enhanced shoot nitrate levels by 1.2-fold and shoot and root free amino acid levels by 3.8-fold and 4.7-fold, respectively (Figure 3). Shoot soluble protein levels were 35% lower in sulfate-deprived plants compared to sulfate-sufficient plants (Figure 3).

Sulfate is required for cysteine synthesis and, therefore, S deficiency hampers protein synthesis [3]. This hampering may not only lower protein contents, but it may also result in an accumulation of nitrate and (non-S-containing) free amino acids, since these are precursors for protein synthesis [3]. The impacts of sulfate deprivation on nitrate, free amino acid and soluble protein content were alleviated by H_2_S fumigation, indicating again that barley used atmospheric H_2_S for cysteine and subsequently protein synthesis (Figure 3). H_2_S fumigation of sulfate-sufficient plants did not affect nitrate, free amino acid and soluble protein levels (Figure 3).

### 2.3. Impact of Sulfate Deprivation and H_2_S Fumigation on Sulfate Uptake and Reduction

**Figure 4:**
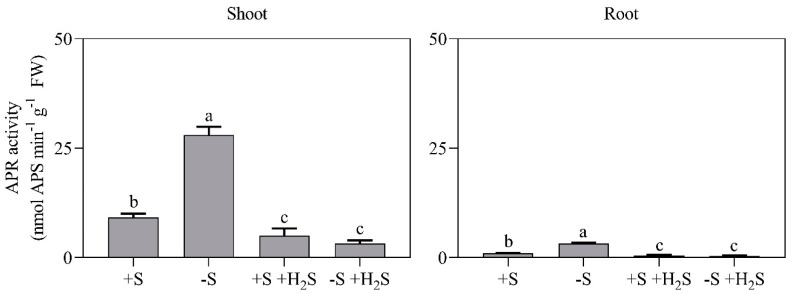
APR activity in barley shoots and roots in the presence and absence of a H_2_S and sulfate supply. For experimental details, see the legend of Figure 1. Data represent the average of 3 measurements with 3 plants per measurement (±SD). Significant differences between treatments are indicated with different letters (*P* ≤ 0.05; two-way ANOVA; Tukey’s HSD test as a post-hoc test).

**Figure 5:**
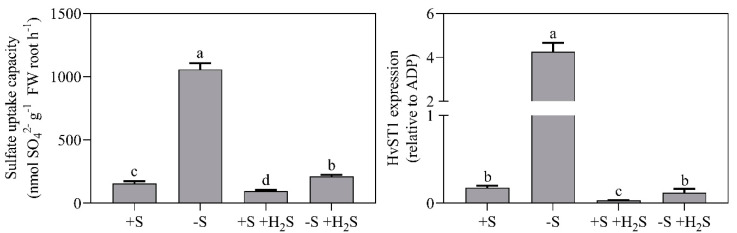
Sulfate uptake capacity and expression of the HvST1 transporter in barley roots in the presence and absence of a H_2_S and sulfate supply. For experimental details, see the legend of Figure 1. Data represent the average of 7 (sulfate uptake capacity) and 3 (HvST1 expression) measurements with 3 plants per measurement (±SD). Significant differences between treatments are indicated with different letters (*P* ≤ 0.05; two-way ANOVA; Tukey’s HSD test as a post-hoc test).

APR activity was approximately 10-fold higher in the shoot than root under all treatments, which indicated that in barley, sulfate reduction primarily takes place in shoot chloroplasts (Figure 4). Sulfate deprivation enhanced shoot and root APR activity by 2.6-fold (Figure 4). The specific APR activity of the shoot was even 3.0-fold enhanced, since sulfate deprivation decreased shoot soluble protein content (Figure 3 and Figure 4). In line with previous observations [11,12], sulfate deprivation also resulted in a higher expression and activity of the root sulfate transporters. The expression of HvST1, a high-affinity root sulfate uptake transporter (K_m_ 6.9 µM sulfate) [11], and the root sulfate uptake capacity were, respectively, 24- and 7-fold higher in sulfate-deprived plants than in sulfate-sufficient plants (Figure 5).

H_2_S fumigation of sulfate-sufficient plants resulted in a 50% decreased shoot and root APR activity (Figure 4). Moreover, it resulted in an 80% and 40% decrease in the root’s HvST1 expression and the sulfate uptake capacity, respectively (Figure 5). Apparently, barley switched from rhizospheric sulfate to atmospheric H_2_S as S source for biomass production. However, since H_2_S fumigation resulted in an enhanced shoot sulfate content (Figure 2), root sulfate uptake may, upon H_2_S fumigation, not be strictly regulated in barley (viz. strictly tuned to the crop’s S demand for growth) [4,13].

H_2_S fumigation of sulfate-deprived plants decreased shoot and root APR activity by approximately 35% (Figure 4). Moreover, it largely alleviated the impacts of sulfate deprivation on the expression and activity of the root’s sulfate transporters (Figure 5). The sulfate uptake capacity of sulfate-deprived fumigated plants was only slightly (1.4-fold) higher than that of sulfate-sufficient non-fumigated plants (Figure 5).

It deserves mentioning here that the impact of S supply on the sulfate uptake capacity mimicked its impact on shoot and root Mo contents (Table 1; Figure 5). Sulfate deprivation resulted in an enhanced sulfate uptake capacity and Mo content, which were alleviated by H_2_S fumigation (Table 1; Figure 5). Molybdate is a structural analogue of sulfate and thus the sulfate transporters in barley may be involved in molybdate uptake [9].

Since atmospheric H_2_S is directly incorporated into cysteine in shoots and since H_2_S fumigation strongly affected the activity of APR and the expression and activity of the sulfate transporters in both sulfate-sufficient and sulfate-deprived plants, our findings indicate that these enzymes are controlled by signals originating in the shoot. Moreover, our observations indicate that the in-situ sulfate concentration in the rhizosphere hardly controls these enzymes.

The nature of the shoot-derived regulatory signals remains ambiguous. Although Vidmar and co-workers [12] suggested glutathione to regulate the sulfate transporters in barley, in the current study glutathione levels did not correlate with the HvST1 expression, sulfate uptake capacity, or APR activity (Figure 2, Figure 4 and Figure 5). This discrepancy may be understood by presuming that variation in glutathione content is accompanied with variation in the content of other metabolites that actually regulate the sulfate transporters.

Sulfate and metabolites from nitrate assimilation (e.g., nitrate and free amino acids) have also been assumed to regulate the sulfate transporters and APR [3]. N and S are needed for cysteine synthesis and thus the rates of nitrate and sulfate assimilation are tuned to each other [3]. However, sulfate, nitrate and free amino acid contents did not clearly correlate with the HvST1 expression, sulfate uptake capacity, or APR activity (Figure 2, Figure 3, Figure 4 and Figure 5). The absence of such correlations makes it tempting to speculate that the activity and expression of the sulfate transporters and APR are instead merely regulated by the sink strength of the shoot for organic sulfur (viz. source-sink dynamics) [4].

### 2.4. Impact of Sulfate Deprivation and H_2_S Fumigation in Barley versus Other Species

In the present research, the interaction between atmospheric H_2_S and rhizospheric sulfate nutrition was analyzed in barley. Previously, this interaction has detailly been analyzed in species from the genus *Brassica* [14,15,16,17,18]. The current data show that this interaction and, therefore, the regulation of sulfate metabolism, partly differs between barley and *Brassica*. Sulfate deprivation of *Brassica* increased the expression of the sulfate uptake transporters, which was associated with a strongly enhanced sulfate uptake capacity [15,18]. Sulfate deprivation of *Brassica* also enhanced the expression and activity of APR in shoots and roots [14,16,17]. Finally, in *Brassica* it resulted in a lower shoot-to-root ratio [14,18]. Notably, sulfate deprivation of barley hardly affected the shoot-to-root ratio (Figure 1). In barley, the shoot-to-root ratio is approximately 2.5, whereas in *Brassica* it is approximately 6 (at an ample sulfate supply; Figure 1) [18]. Therefore, barley constitutively has relatively more roots than *Brassica* and, upon sulfate deprivation, it may increase its sulfate uptake capacity without the need to allocate resources to root synthesis.

H_2_S fumigation of sulfate-sufficient *Brassica* downregulated shoot and root APR activity as well as the expression and activity of the root sulfate uptake transporters [4,14,15,17]. Clearly, similar to barley, *Brassica* switched from rhizospheric sulfate to atmospheric H_2_S as S source. Thus, in both plants sulfate utilization is controlled by signals originating in the shoot. In line with this, cutting of the shoot of curly kale (*Brassica oleracea*) rapidly decreased the plants’ sulfate uptake capacity. Similar to barley, in *Brassica* the nature of the shoot-derived regulatory signals remains ambiguous [4].

Dissimilar to *Brassica*, in barley H_2_S fumigation resulted in an enhanced shoot sulfate content (Figure 2) [15]. Notably, in spruce (*Picea abies*) spinach (*Spinacia oleracea*), red clover (*Trifolium pratense*) soybean (*Glycine max*), common bean (*Phaseolus vulgaris*) and onion (*Allium cepa*) fumigation with an atmospheric H_2_S level that is sufficient to cover the S requirement for growth, also resulted in an enhanced sulfate content [4,13,19,20]. Thus, potentially, upon H_2_S fumigation in most plants, sulfate uptake is less strictly regulated than in *Brassica* (viz. less strictly tuned to the plant’s S demand for growth).

Fumigation of sulfate-deprived *Brassica* with ≥0.06 µL L^−1^ H_2_S alleviated the sulfate-deprived decreases in biomass production and in cysteine and glutathione contents [14,15,18]. Additionally, it alleviated the enhanced expression of APR upon sulfate deprivation. However, dissimilar to barley, in *Brassica* H_2_S fumigation hardly affected the sulfate-deprived enhancement of the expression and activity of the root sulfate uptake transporters (Figure 5) [15,16,17,18]. Moreover, it hardly affected the sulfate-deprived decrease in the shoot-to-root ratio [15,16,17,18]. This indicates that, dissimilar to barley, in *Brassica* sulfate uptake is, besides by shoot-derived signals, controlled by the in-situ sulfate concentration in the rhizosphere. It has been assumed that, upon sulfate deprivation, *Brassica* forms special roots that are typified by a high sulfate transporter expression and activity, which cannot be modified by the plant’s internal sulfur status [17]. Since H_2_S exposure of sulfate-deprived barley strongly alleviated the increased expression and activity of the sulfate transporters, apparently barley hardly forms such roots upon sulfate deprivation.

## 3. Materials and Methods

### 3.1. Plant Material and H_2_S Fumigation

Barley (cv. KWS Irina; Wiersum Plantbreeding; Winschoten; The Netherlands) was germinated on boxes, containing 15 l aerated tap water, in a climate-controlled room with temperature, humidity, light intensity and light duration settings identical to those described in [7]. After 7 days, the seedlings were transferred to stainless-steel boxes (60 plants per box) holding 13 l aerated 50% Hoagland nutrient solutions. Nutrient solutions either contained 1 mM sulfate (+S; sulfate-sufficient; solution’s composition being 2.5 mM CaCl_2_, 2.5 mM KCl, 0.5 mM KH_2_PO_4_, 1 mM MgSO_4_, 3.75 mM NH_4_NO_3_, 23.4 µM H_3_BO_3_, 4.8 µM MnCl_2_, 0.48 µM ZnSO_4_, 0.16 µM CuSO_4_, 0.26 µM Na_2_MoO_4_ and 45 µM Fe^3+^EDTA) or 0 mM sulfate (-S; sulfate-deprived; all sulfate salts replaced by chloride salts). The boxes were placed in 50 L cylindrical stainless-steel cabinets (0.6 m diameter) with a polymethyl methacrylate top with temperature, humidity, light intensity and light duration settings identical to those described in [7]. In two independent (identical) experiments, the plants were exposed to 0 or 0.6 µL L^−1^ atmospheric H_2_S (for details on H_2_S application, see [7]).

After 7 days of treatment, plants were harvested 3 h after the start of the light period. After the root was rinsed in ice-cold de-mineralized water (3 × 20 s), the shoot and root were separately weighted. The shoot and root biomass productions were calculated by subtracting the initial, pre-treatment weight from the weights at harvest. For the measurement of the dry matter content and the mineral nutrient composition, shoots and roots were dried overnight at 80 °C. For the determination of the S and N metabolite content, APR activity and sulfate transporter expression, shoots and roots were frozen at −80 °C.

### 3.2. Mineral Nutrient Content

The mineral nutrient content of shoots and roots was determined from dried plant material, that was pulverized using a Retsch Mixer-Mill (Retsch type MM2, Haan, Germany). Total N content was measured via the Dumas procedure, using an automated elemental analyzer (model EA 1110; Interscience, New York, NY, USA) with Eager 200 for Windows [21]. The levels of other minerals were analyzed via inductively coupled plasma mass spectrometry (ICP-MS) with an Agilent 7700 ICP-MS (Agilent Technologies, Santa Clara, CA, USA) [22].

### 3.3. Sulfur and Nitrogen Metabolite Content

Water-soluble non-protein thiols were isolated from freshly harvested plant material and their content was quantified colorimetrically according to [23]. Sulfate and nitrate were isolated from frozen plant material according to [24] and their contents were quantified with ion chromatography (IC) as described in [25]. Free amino acids and soluble proteins were isolated from frozen plant material and quantified as described by [26].

### 3.4. APR Activity and Sulfate Uptake Capacity

For the determination of APR activity, frozen shoots and roots were ground with liquid N_2_. The resulting plant powder was homogenized in 1 mL 50 mM K_2_PO_4_ buffer (pH 8.0) that contained 30 mM Na_2_SO_4_, 500 µM AMP and 10 mM DTE. After centrifugation, APR activity was measured as described in [27]. For the determination of the sulfate uptake capacity, plants were incubated for 1 h on a 25% Hoagland nutrient solution that was labeled with 500 µM ^35^S-sulfate (2 MBq L^−1^), after which the sulfate uptake capacity was assessed following [16].

### 3.5. Expression of HvST1

To determine the expression of HvST1, total RNA was isolated from frozen plant material, made free of genomic DNA and transcribed into cDNA as described by [18]. The acquired cDNA samples were diluted 1:50 in distilled water. During quantitative PCR (qPCR), ADP-ribosylation factor 1-like protein (ADP) was used as reference gene, since its expression is stable across several environmental conditions [28]. Primers for ADP and HvST1 were retrieved from [28] and [29], respectively. The qPCR reaction mixture contained 2 µL 1:50 diluted cDNA, 12.5 µL 2x Bio-Star qPCR-Mastermix SYBR Blue (GeneON GmbH; Ludwigshafen; Germany), 0.75 µL ROX (GeneON GmbH), 0.75 µL of each primer (10 µM stock) and 8.25 µL deionized water. Reactions were run in triplicate on an Applied Biosystems 7300 Real Time PCR system (Applied Biosystems, Foster City, CA, USA) with an initial denaturation of 5 min at 95 °C, followed by 50 cycles of 15 s denaturation at 95 °C, 15 s annealing at 60 °C and 30 s elongation at 72 °C. The program was finished by denaturation from 65 °C to 95 °C to generate melting curves (to verify a-gene-specificity of the primers). The LinRegPCR software (version 2014.2; Heart Failure Research Centre; Amsterdam, The Netherlands) was used to baseline-correct the qPCR data, after which the initial number of gene transcripts (N_0_) in a sample was determined with the mean PCR efficiency per primer set (which was between 95% and 100%) [30,31,32]. For the calculation of the relative expression level of HvST1, the N_0_ value of HvST1 was divided by the N_0_ value of ADP.

### 3.6. Statistical Analyses

GraphPad Prism (version 8.4.2; GraphPad Software, San Diego, CA, USA) was used for statistical analyses. Treatment means were compared using a two-way analysis of variance (ANOVA) with a Tukey’s HSD test as post-hoc test at the *P* ≤ 0.05 level.

## 4. Conclusions

H_2_S fumigation alleviates S deficiency in barley, which could grow with H_2_S as the only S source. Moreover, in barley there was a strong interaction between the metabolism of atmospheric H_2_S and rhizospheric sulfate. H_2_S exposure downregulated APR activity and the expression and activity of the root sulfate transporters. Clearly, similar to observations in *Brassica*, in barley shoot-derived signals regulate sulfate utilization. However, dissimilar to *Brassica*, in barley the in-situ sulfate concentration in the rhizosphere hardly regulates sulfate utilization. The nature of the shoot regulatory signals needs to be elucidated in further studies.

## Figures and Tables

**Table 1:** Mineral nutrient content of barley shoots and roots in the presence and absence of a H_2_S and sulfate supply. For experimental details, see the legend of Figure 1. Data represent the average of 3 measurements with 6 plants per measurement (±SD). Significant differences between treatments are indicated with different letters (*P* ≤ 0.05; two-way ANOVA; Tukey’s HSD test as a post-hoc test).

Mineral Nutrient Content	0 µL L^−1^ H_2_S		0.6 µL L^−1^ H_2_S	
(µmol g^−1^ DW)	+S	−S	+S	−S
*Shoot*				
Calcium	103 ± 10 a	84 ± 3 a	100 ± 29 a	81 ± 8.0 a
Copper	0.29 ± 0.03 a	0.25 ± 0.01 a	0.29 ± 0.05 a	0.25 ± 0.03 a
Iron	1.92 ± 0.12 a	1.65 ± 0.07 a	2.06 ± 0.45 a	1.72 ± 0.20 a
Magnesium	81 ± 7 a	76 ± 3 a	88 ± 16 a	71 ± 8 a
Manganese	0.73 ± 0.06 ab	0.66 ± 0.03 ab	0.83 ± 0.14 a	0.59 ± 0.05 b
Molybdenum	0.04 ± 0.00 c	0.25 ± 0.01 a	0.04 ± 0.01 c	0.19 ± 0.03 b
Nitrogen	3521 ± 150 a	3216 ± 61 b	3412 ± 71 ab	3418 ± 69 ab
Phosphorus	192 ± 9 b	250 ± 11 a	204 ± 34 ab	180 ± 22 b
Potassium	1758 ± 79 a	1637 ± 94 a	1852 ± 205 a	1570 ± 161 a
Sulfur	99 ± 4 b	36 ± 2 c	163 ± 16 a	100 ± 12 b
Zinc	0.63 ± 0.07 ab	0.77 ± 0.05 a	0.67 ± 0.11 ab	0.50 ± 0.03 b
*Root*				
Calcium	26 ± 6 a	38 ± 4 a	27 ± 3 a	21 ± 1 a
Copper	2.05 ± 0.43 a	1.13 ± 0.02 b	1.83 ± 0.15 a	1.45 ± 0.11 ab
Iron	2.92 ± 1.14 a	3.50 ± 0.46 a	2.34 ± 0.05 a	2.78 ± 0.12 a
Magnesium	28 ± 6 a	31 ± 3 a	30 ± 6 a	25 ± 1 a
Manganese	2.21 ± 0.37 a	1.17 ± 0.08 b	2.36 ± 0.21 a	1.34 ± 0.11 b
Molybdenum	0.14 ± 0.02 b	0.42 ± 0.07 a	0.17 ± 0.03 b	0.18 ± 0.01 b
Nitrogen	3231 ± 133 a	2917 ± 114 a	3174 ± 182 a	3152 ± 121 a
Phosphorus	149 ± 21 a	100 ± 8 b	153 ± 12 a	134 ± 16 ab
Potassium	842 ± 103 a	662 ± 21 b	844 ± 42 a	719 ± 56 ab
Sulfur	82 ± 8 a	28 ± 0 b	94 ± 9 a	44 ± 4 b
Zinc	0.59 ± 0.12 a	0.40 ± 0.05 b	0.57 ± 0.04 ab	0.47 ± 0.03 ab
